# Comparison of the Dynamic Cut‐Out Failure Modes of Common Proximal Femoral Fixation Devices Using a Mesh‐Free Computational Method

**DOI:** 10.1002/jor.70159

**Published:** 2026-02-10

**Authors:** Erica Ueda Boles, Sloan Kulper, Katie Whiffin, Marilyn Janice Oentaryo, Kerstin Schneider, Frankie Leung, Christian X. Fang

**Affiliations:** ^1^ Lifespans Ltd. Hong Kong China; ^2^ Department of Orthopaedics & Traumatology, LKS Faculty of Medicine The University of Hong Kong Hong Kong China; ^3^ School of Biomedical Engineering The University of Hong Kong Hong Kong China; ^4^ Orthopaedic Surgeon Zurich Switzerland

**Keywords:** cut‐out, dynamic, internal fixation, mesh‐free, migration, proximal femur, simulation

## Abstract

Migration and cut‐out are commo n failures observed in internal fixation of intertrochanteric fractures. This study compares the performance of four typical devices under dynamic loading using benchtop and computer‐simulated fracture models. The dynamic hip screw (DHS), Gamma3, proximal femoral nail anti‐rotation (PFNA‐II), and TFN‐Advanced (TFNA) were inserted into a solid rigid 10 pounds per cubic foot (PCF) polyurethane bone foam mimicking a reduced unstable intertrochanteric fracture model. Static and dynamic loading tests using a double‐peak loading curve based on natural walking gait were conducted. The blade devices had higher resistance to the onset of implant migration under physical dynamic loading in comparison to the screw devices. However, as dynamic loading progressed, no implant showed clear superior performance. Mesh‐free simulations of the physical tests were then conducted to visualize stress and failure patterns within the bone foam during migration and cut‐out, using an experimentally validated porous foam material model. The mean concordance correlation coefficient between load to cut‐out for the physical and simulated tests was 0.953 and 0.858 under static and dynamic loading, respectively. Under simulated dynamic loading, the volumes of yielding and failed bone foam were 15%, 32%, and 25% higher for Gamma3, PFNA‐II, and TFNA than for DHS, suggesting that devices with similar forces at cut‐out may produce relatively large differences in the volume of damaged bone tissue. Accurate simulation of material damage during dynamic loading may offer a useful alternative method for differentiating device performance in support of clinical decision‐making around implant selection.

## Graphical abstract

Migration and cut‐out of DHS, Gamma3, PFNA‐II, and TFNA were compared under dynamic loading in a stable intertrochanteric fracture benchtop foam model and a mesh‐free simulation. No significant differences in force at cut‐out were observed, but simulation revealed that the volumes of damaged bone foam were 15%, 32%, and 25% higher for the Gamma3, PFNA, and TFNA than for the DHS, suggesting that devices with similar forces at cut‐out may produce large differences in the volume of damaged bone tissue.

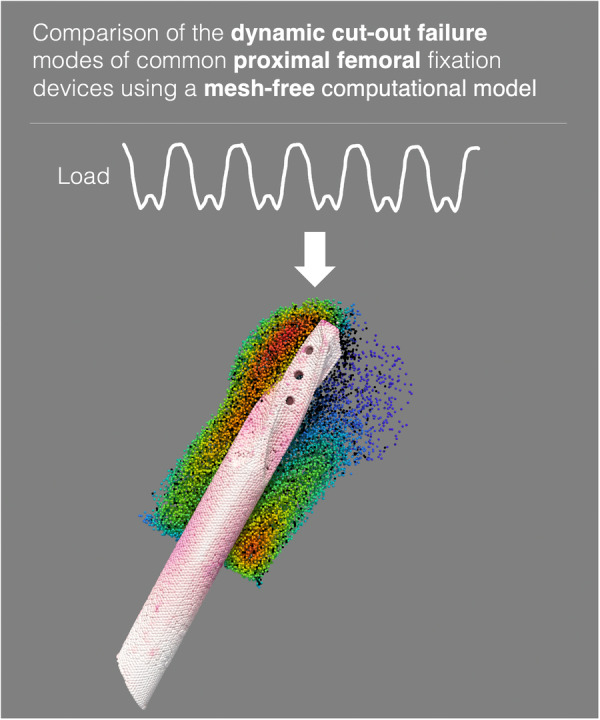

## 1 Introduction

Migration and cut‐out failures of internal fixation devices for intertrochanteric fracture repair are a major healthcare problem in the ageing population worldwide [1, 2, 3, 4, 5, 6]. Osteoporotic fractures affect more than 50% of women and 25% men during their lifetimes [6, 7], and the incidence of hip fractures is expected to grow to > 6.3 million per annum by the year 2050 [8, 9, 10, 11]. Postsurgical migration and cut‐out of intertrochanteric screws or blades from within the femoral head can lead to loss of reduction, varus collapse, and trauma to adjacent tissue to the extent that surgical revision is required [12]. The rate of cut‐out in the current clinical literature is reported to range between 2.8% and 8.7% [8, 9, 11], while the rate of all complications including implant migration as a comorbidity ranged between 3.7% and 10.2% [9, 10, 11, 13, 14].

Clinical decision‐making when selecting the fixation device at the lowest risk of migration and cut‐out is hindered by a lack of clear supporting evidence, partly due to heterogeneity and inconsistency in reporting [8, 9, 11, 13, 14]. This is particularly true for unstable trochanteric fractures. The typical surgical treatment of intertrochanteric fractures is osteosynthesis with either intramedullary (e.g., Gamma3, proximal femoral nail anti‐rotation [PFNA‐II], TFN‐Advanced [TFNA]) or extramedullary devices (e.g., dynamic hip screw [DHS]) [10, 13, 15]. In common clinical practice, intramedullary devices are generally preferred for unstable fractures despite their higher costs. Given the weakness of evidence supporting this trend [16, 17], very high usage rates of intramedullary devices in recent years (e.g., up to 90% in the United States) [9] may indicate overuse of this device type. Biomechanical investigations of unstable trochanteric fracture fixation are therefore still relevant to clinical practice as they may provide supplementary evidence to support implant selection, reducing the risk of postsurgical failure as well as the cost of treatment.

Simulation represents a unique pathway for generating biomechanical evidence by visualizing and quantifying internal stress, strain, and damage during migration and cut‐out. Cut‐out failure typically occurs after several 1000 or more physiological loading cycles [18, 19], with implant migration gradually accruing in a process of dynamic aseptic loosening. While numerous benchtop dynamic loading studies of unstable trochanteric fractures have been performed in cadaveric [20, 21, 22, 23] and surrogate bone (polyurethane foam) [24, 25, 26, 27], the relatively few simulation studies in the literature have generally focused on static loading conditions [28, 29, 30, 31, 32, 33, 34, 35, 36, 37] or several loading cycles [38, 39]. None attempted to match or predict migration and cut‐out resulting from thousands or more loading cycles. All unstable trochanteric fracture simulation studies in the literature were performed using a mesh‐based modeling approach, such as the finite element method (FEM). The accuracy of mesh‐based simulation generally declines as material stress exceeds the plastic limit, though it can be extended to include some high‐strain phenomena during implant subsidence in bone [40, 41]. Conversely, mesh‐free modeling [42] is a newer approach most often used for high‐strain applications [43], making it well suited to simulation of implant migration and cut‐out.

We present a novel mesh‐free computational method to predict the migration and cut‐out of unstable trochanteric fracture fixation devices under thousands of physiologically relevant loading cycles. Simulations were performed for four types of common femoral fixation devices (DHS, Gamma3, PFNA‐II, and TFNA) in porous 10 pounds per cubic foot (PCF) rigid polyurethane foam under static and dynamic loading. Benchtop tests were used for validation. Previous studies using mesh‐free [42] simulation have examined large deformations and material damage at the bone‐implant interface [44], trabecular bone [45, 46], and cortical bone biomechanics [47], bone remodeling [48], spinal biomechanics [49], and cement infiltration [50] of the mandible. To the authors’ knowledge, this study is the first to present a dynamic simulation model of unstable trochanteric fractures. Furthermore, by comparing the force at failure and quantifying the volume of damaged bone tissue at cut‐out, this study provides evidence to support clinical selection of fixation devices for unstable intertrochanteric fracture repair.

## 2 Methods

A series of benchtop experiments were conducted to evaluate the relative likelihood of migration and cut‐out of four common proximal femoral internal fixation devices (DHS, Gamma3, PFNA‐II, and TFNA) under dynamic loading conditions. Implants were inserted into stable intertrochanteric osteoporotic fracture models of the femur consisting of solid rigid polyurethane bone foam enclosed in a steel cortical shell. Static loading experiments were also performed to provide calibration data for the material models used in the simulations. Mesh‐free simulations of the physical tests were then conducted to visualize stress and failure patterns within the bone foam during migration and cut‐out.

### 2.1 Static and Dynamic Loading Experimentation Setup

**Figure 1:**
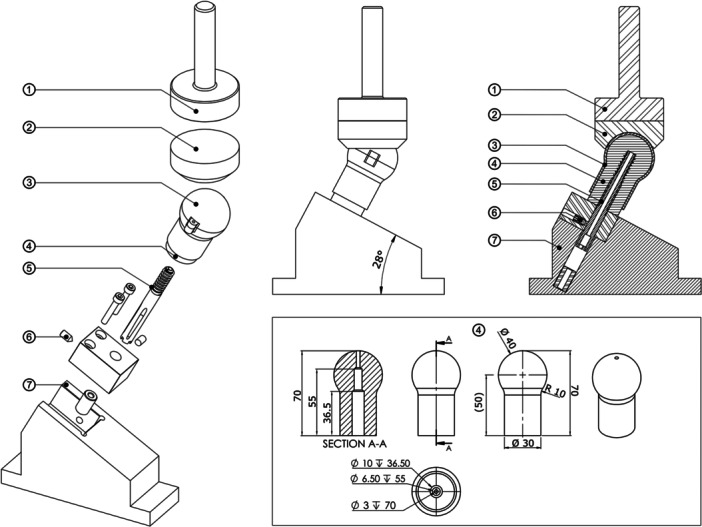
Illustration of physical experiment setup, consisting of: ① hydraulic press loading fixture of stainless steel, ② sliding acetabulum of stainless steel, ③ proximal femoral shell of stainless steel, ④ proximal femur of solid rigid 10 PCF polyurethane bone foam (shown here as prepared with a pilot hole for the Gamma3), ⑤ proximal femoral fixation device (shown here as a Gamma3), ⑥ anti‐rotation set screw, and ⑦ base fixture of stainless steel, clamped to load cell (fixed, not shown). Dimensions in millimeters.

**Figure 2:**
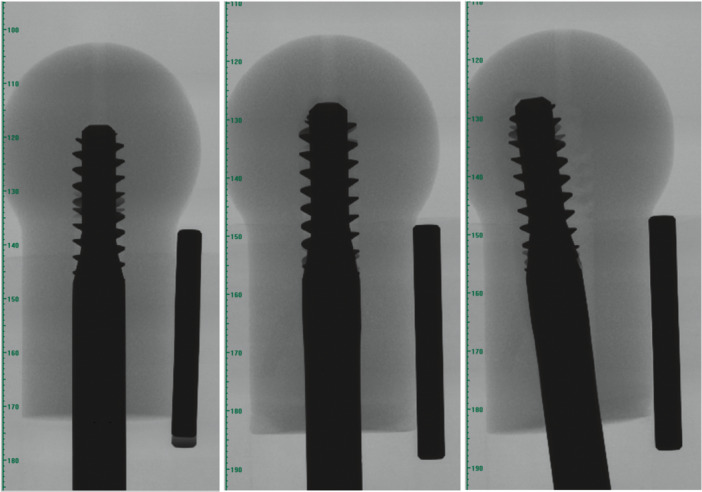
Insertion of Gamma3 device into foam femur under x‐ray with steel rod of known dimensions for reference, before dynamic loading test (left), and after dynamic loading test in medio‐lateral view (center) and anterior‐posterior view (right).

A stable intertrochanteric osteoporotic fracture model of the femur was fabricated for benchtop testing (Figure 1). Osteoporotic bone foam models of the proximal femur (*n* ≥ 4 for each device tested) were machined on a lathe from blocks of bone foam (10 PCF solid rigid polyurethane bone foam, SKU #1522‐01, 130 mm × 180 mm × 40 mm, Pacific Research Laboratories, USA), resulting in a final overall length of 70 mm and Ø 40 mm femoral head. The direction of rise was parallel to the foam block thickness, and all foam femurs were machined with the rise direction parallel to the anterior–posterior axis. Pilot holes were prepared in the proximal femur models using the instrument set and surgical technique provided by the implant manufacturer for each device. Four proximal femoral internal fixation devices ([DHS, DePuy Orthopaedics Inc., USA], Gamma3 [Stryker Corporation, Michigan, USA], [PFNA‐II, DePuy Orthopaedics Inc., Massachusetts, USA], and Trochanteric Fixation Nail Advanced [TFNA, DePuy Orthopaedics Inc., Massachusetts, USA]) were inserted to a tip apex distance of 15 mm to the joint (30 mm total nominal tip apex distance (TAD) in anterior–posterior plus medio–lateral views). Insertion depth was verified by x‐ray (Figure 2) (Bruker Skyscan 1276, Bruker Corporation, Massachusetts, USA). This is shallower than the typically recommended total TAD of less than 25 mm, thus, the implant insertion depth in this study tests nonoptimal (but clinically realistic) depth positioning of the implant [51]. A cortical shell of polished 316L stainless steel was fabricated to enclose the bone foam femurs, distribute load during migration and cut‐out testing, and allow sliding against the acetabulum to allow for varus rotation of the foam femur [52, 53, 54]. The steel shell did not enclose the distal 7.5 mm of the foam femur to allow for varus collapse of the distal end of the foam femur to simulate a reduced unstable pertrochanteric fracture with deficient posteromedial neck support. Implants were allowed to slide (but not rotate) within a base fixture of stainless steel to simulate reduction of the fracture, such that the flat distal surface of the foam femur rested on the top loading surface of the fixture (angled 28° above horizontal). This angle was chosen based on the DHS construct having a 135° angle, and thus fixing the screw at a 62° angle from vertical simulates a clinical 17° angle of peak walking force vector in the frontal plane acting towards the center of the femoral head [55]. Cannulated inserts were fabricated for each implant type with an adjustable set screw such that the implants could slide axially, but not rotate, within the base fixture. An acetabulum of polished 316L stainless steel was placed superior to the cortical shell and loaded via a flat loading fixture to allow translation of the acetabulum in the horizontal transverse plane during loading. The loading fixture was mounted to an MTS 858 Mini Bionix (MTS Inc., Minnesota, USA) hydraulic loading machine, and the base fixture was clamped to a fixed 10 kN load cell.

**Figure 3:**
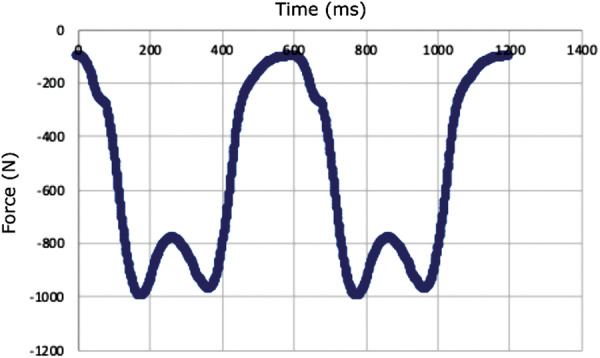
First two cycles of the double‐peak loading curve (force in the negative z‐direction measured in Newtons vs. time in milliseconds) used during physical dynamic testing.

The force detected by the MTS was zeroed, then the loading fixture was slowly lowered until just touching the acetabulum such that the force detected was less than 5 N. Static or dynamic loading tests were then performed. Static loading tests were performed for calibration on the DHS and Gamma3 (*n* ≥ 4 per device), during which the constructs were compressed in the inferior direction until cut‐out occurred (defined as displacement of 7 mm). The static loading program performed an initial axial compression ramp from ~5 to 50 N in 5 s, followed by displacement control at 2 mm/min to 7 mm of vertical displacement. The force–displacement curves were plotted for each sample. Dynamic loading tests were then performed for all four devices (*n* ≥ 4 per device) implanted in new foam femurs to the same insertion depth as the static tests. The dynamic loading conditions were adapted from the resultant forces measured in the hip joint during walking from Orthoload (AVER75 data set) [55]. The dynamic loading program performed an initial non‐destructive, quasi‐static, axial compression ramp from ~5 to 100 N in 15 s [56]. Then cyclic loading began using a double‐peak loading curve at 1 Hz with the minimum and maximum force ranging from 100 to 1000 N, respectively, for the first cycle, and the maximum force increased 0.1 N per cycle with the minimum force kept at 10% of the maximum force (Figure 3) [57, 58, 59]. Dynamic loading continued until 7 mm of vertical displacement was reached. The cycle and corresponding maximum force level required to first reach a given displacement (in 0.5 mm intervals) was determined for each sample.

### 2.2 Mesh‐Free Simulation Setup

Mesh‐free simulations of the static and dynamic tests were conducted to visualize stress and failure patterns within the bone foam during migration and cut‐out. All material models employed either rigid‐body or compressible neo‐Hookean [60] constitutive models, using thresholds of maximum principal strain (for metallic bodies) or maximum principal stress (for bone foam) to determine failure. Neither viscoelastic nor viscoplastic properties were included.

**Figure 4:**
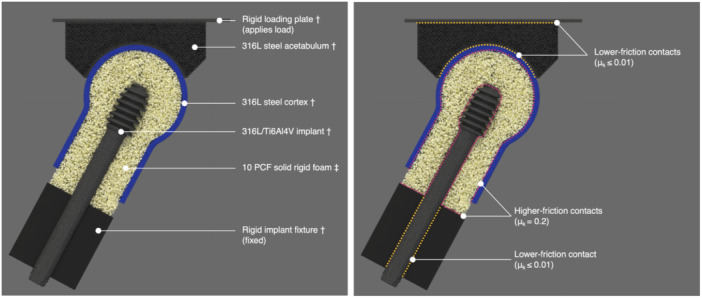
Cross‐sectional view of the mesh‐free simulation setup, showing materials and boundary conditions (left) and contacts (right) of test fixtures, implant, and bone foam. ^†^Implant and fixtures geometry modeled as volumetric point clouds with mesh‐fitting localized refinement performed to achieve smooth bearing surface (nominal particle resolution of 160–640 µm). ^‡^Bone foam geometry modeled as porous point cloud with quasi‐random distribution of 400 µm particles (mean BV/TV of 0.14, proportional to 10 PCF solid rigid PU foam).

Solid model representations of the implants and test fixtures were prepared in CAD (SolidWorks v.2023–2024 [Dassault Systèmes, Vélizy‐Villacoublay, France], Creo v.9.0 [PTC Inc., Massachusetts, USA], and Rhinoceros 8 [Robert McNeel & Associates, Washington, USA]) within the tolerance of ISO 2768 (fine). These models were then meshed in Salome v.9.13 (EDF‐CEA, Paris, France), and volumetric point clouds were generated in Alfonso v.Y‐20240311 (Lifespans Ltd., Hong Kong) using mesh‐fitting localized refinement to achieve smooth bearing surfaces (nominal particle cross‐section of 160–640 µm, see Figure 4). The mechanical properties of metallic components (implants and test fixtures) were assigned according to the published standards for each respective material (Table 1) using a material model and assumed loading rate (2 m/s) that was experimentally validated in a prior three‐point bending study (see Figure S1).

**Table 1:** Material properties assigned in mesh‐free simulation models of implant, fixtures, and bone foam.

**316L stainless steel (cold worked) per DIN 1.4404/UNS S31603**		
Young's modulus	*E*	193 GPa
Poisson's ratio	*v*	0.27
Yield stress	*σ*_*y*_	800 MPa
Elongation at break	*ε*	0.6
Hardening modulus	*H*	500 Pa
**Type 23 wrought titanium (Ti6Al4V) UNS R56401/Ti6Al7Nb UNS R56700**		
Young's modulus	*E*	110 GPa
Poisson's ratio	*v*	0.31
Yield stress	*σ*_*y*_	860 MPa
Elongation at break	*ε*	0.1
Hardening modulus	*H*	3.3 GPa
**10 PCF solid rigid PU foam ASTM F1839‐08 (properties of overall porous structure)**		
Young's modulus	*E*	58 MPa
Yield stress	*σ*_*y*_	2.2 MPa
Volume fraction (ratio of PU polymer to total volume of porous structure)	VF	0.14
**10 PCF solid rigid PU foam ASTM F1839‐08 (properties of compact polymer portion of porous structure)**		
Young's modulus	*E*_sd_	493 MPa*†
Yield stress (static)	*σ*_ys_	18.70 MPa*
Ultimate stress (static)	*σ*_us_	19.64 MPa*
Yield stress (dynamic)	*σ*_yd_	16.83 MPa†
Ultimate stress (dynamic)	*σ*_ud_	17.67 MPa†
Poisson's ratio	*V*	0.30
Hardening modulus	*H*	0

*For static tests, an 8.5x correction coefficient was uniformly applied to the elastic modulus, yield stress, and ultimate stress values of the 10 PCF foam, as determined by prior validation studies (see Supporting File). †For dynamic tests, yield and ultimate stress values were reduced by 10% to accelerate damage accumulation.

Solid model representations of the foam femurs were prepared in CAD and imported into Alfonso for point cloud generation. Particle generation and simulation of implant, fixtures, and foam may similarly be performed in the DEM/SPH package of Abaqus FEA (Dassault Systèmes, Vélizy‐Villacoublay, France) as demonstrated in prior research [44]. A porous point cloud was generated to fill the boundary of each foam femur with a quasi‐random distribution of mesh‐free particles of polymer and void spaces comparable in both density and homogeneity to 10 PCF solid rigid PU foam. Particle resolution (size) was a uniform 400 µm, and the mean volume fraction of layer of simulated material was 0.14, matching that of 10 PCF foam. For each respective implant type, foam particles with centroids intersecting the implant solid model were removed. Implant insertion was not simulated.

The mechanical properties of 10 PCF foam as published in standards documents [61] are representative of the overall foamed porous structure, rather than the compact solid polymer itself, and thus cannot be directly assigned to the mesh‐free particles in the present study. A correction coefficient was determined during a prior validation study (see Supporting Material) to assign the appropriate material properties to particles of compact solid polymer given the particle size of the present study. For the static tests, this coefficient was applied uniformly to the original Young's modulus and yield and ultimate stresses of the 10 PCF foam material. Contacts and boundary conditions were then defined as shown in Figure 4, with the rigid loading plate moved downward (‐z direction) at a loading rate of 2 m/s until 7 mm displacement was reached.

Given that the purpose of the dynamic simulation study was to predict the force (rather than the cycle count) at cut‐out for each device under a monotonically increasing load, these simulations used a single loading cycle with boundary conditions and contacts identical to the static simulations. Yield and ultimate stress thresholds for the foam material model were reduced by 10% from the values of the static simulations to accelerate damage accumulation. The physical dynamic test results of the DHS were used to calibrate the value of this coefficient, which was then applied to the foam models used in the dynamic simulations of the three remaining devices. Yielding and failed particles were extracted to quantify and compare the volumes of bone foam damaged by each respective device during loading. To help visualize differences between the locations of damaged bone foam, density heatmaps were created in CloudCompare v.2.14.alpha (GPL Software) [62] by calculating the number of neighboring yielding particles within a 6 mm radius.

### 2.3 Statistical Analysis

Initial loading artifacts at low force values (e.g., toe‐in) were removed such that the force values at zero depth were normalized between physical and simulated pairs. Since the physical static Gamma3 test ended just before 7 mm of vertical displacement was reached, a linear extrapolation using the slope from 3 mm onwards was used to estimate the load at 7 mm of displacement for each physical Gamma3 static loading curve. For comparison of the physical static results, a *t*‐test (two‐sample assuming equal variances with alpha = 0.05) was performed using Microsoft Excel Version 16.102.3. For comparison of the physical dynamic results, one‐way analysis of variance (ANOVA) was used to test the difference between the means of the four implant test groups (IBM SPSS Statistics 31.0.1.0, Armonk, New York). If the ANOVA test resulted in a *p*‐value less than the significance level of 0.05 (*p* < 0.05), the Tukey post hoc test was used for pairwise multiple comparison of the implant groups. Prior to the ANOVA test, Levene's Test for Equality of Variances was performed (*p* = 0.482, based on mean), and the symmetry and peakedness of the data for each group was visualized using box‐and‐whisker plots (not shown) to check that the variances and distribution of the different groups were not significantly different. Lin's concordance correlation coefficient (CCC) [63] was used in the present study to compare the similarity between the F‐D curves of physical and simulated testing pairs. This statistical method evaluates the degree to which pairs of observations fall on the 45° line through the origin (i.e., the line of equality). Force‐displacement (F‐D) data from the physical and simulated tests study groups (Table 2) were matched by *D* values, and the CCC values were computed using Microsoft Excel Version 16.102.3. The CCC is calculated as *ρ*_c_ = *ρ* x *C*_b_ (–1 ≤ *ρ*_c_ ≤ 1) where:

**Table 2:** Force at cut‐out.

	Physical	Simulated
Static, DHS	*n* = 5	*n* = 1
Mean (*N*)	2294	2274
Standard deviation (*N*)	174	nil
Static, Gamma3	*n* = 4	*n* = 1
Mean (*N*)	2314	2390
Standard deviation (*N*)	187	nil
Dynamic, DHS	*n* = 7	*n* = 1
Mean (*N*)	2219	2132
Standard deviation (*N*)	98	nil
Dynamic, Gamma3	*n* = 6	*n* = 1
Mean (*N*)	1959	2194
Standard deviation (*N*)	53	nil
Dynamic, PFNA‐II	*n* = 4	*n* = 1
Mean (*N*)	2161	2354
Standard deviation (*N*)	81	nil
Dynamic, TFNA	*n* = 4	*n* = 1
Mean (*N*)	2267	2187
Standard deviation (*N*)	94	nil

*ρ* is the Pearson correlation coefficient, which measures how far each observation deviates from the best‐fit line, and is a measure of precision, and

*C*_b_ is a bias correction factor that measures how far the best‐fit line deviates from the 45° line through the origin and is a measure of accuracy (0 < *C*_b_ ≤ 1; *C*_b_ = 1 when there is no deviation from the 45° line).

A CCC value near +1 indicates strong concordance, a value near −1 indicates strong discordance, and a value near zero indicates no concordance. In the present study, we interpret Lin's CCC in the manner that Pearson's correlation coefficient or intraclass correlation coefficients are commonly interpreted, namely that values ≤ 0.20 indicate “poor” concordance and values ≥ 0.80 indicate “excellent” concordance, between test pairs.

## 3 Results

**Figure 5:**
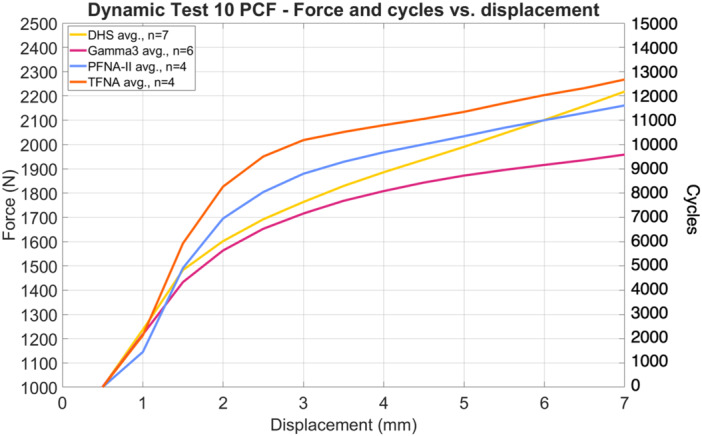
Peak force reached during each loading cycle of physical dynamic testing.

Under physical static loading, the mean forces at cut‐out were 2294 N (*σ* = 174 N) and 2314 N (*σ* = 187 N) for the DHS and Gamma3, respectively (*t*‐test, two‐tailed *p* = 0.87). Under physical dynamic loading, the mean forces at cut‐out were 2219 N (*σ* = 98 N), 1959 N (*σ* = 53 N), 2161 N (σ = 81 N), and 2267 N (*σ* = 94 N) for the DHS, Gamma3, PFNA‐II, and TFNA, respectively (Table 2), with all specimens reaching approximately 9500–12,750 cycles before cut‐out was reached (Figure 5). Analysis using one‐way ANOVA detected that a significant difference exists among the mean maximum force at cut‐out between device types during physical dynamic testing (*F*‐statistic = 14.590, *p* < 0.001). Tukey post hoc pairwise multiple comparison tests detected a significantly different mean maximum force at cut‐out for Gamma3 in comparison to DHS (*p* < 0.001), PFNA‐II (*p* = 0.008), and TFNA (*p* < 0.001), while no significant difference in the mean was detected between these latter three implant groups.

**Figure 6:**
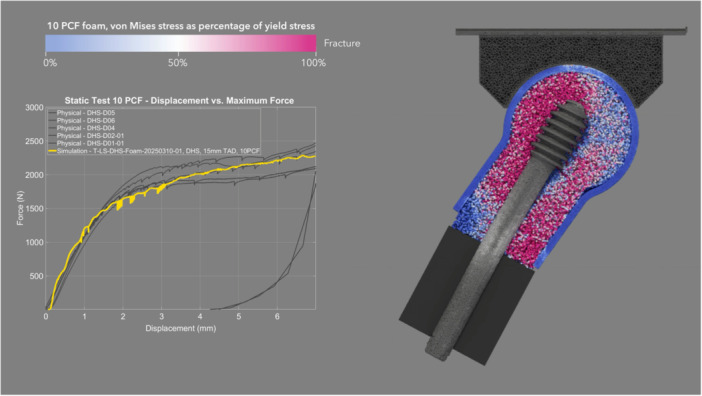
Simulation and physical (gray lines) results of DHS under static loading, cross sectional view through foam proximal femur.

**Figure 7:**
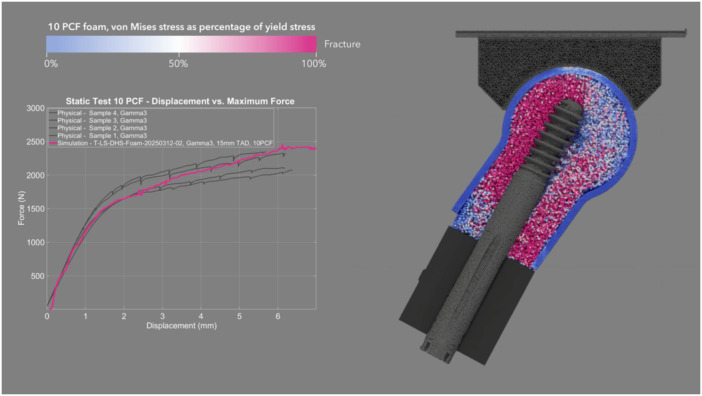
Simulation and physical (gray lines) results of Gamma3 under static loading, cross‐sectional view through foam proximal femur.

The mean CCC between physical and simulated static loading pairs—DHS (Figure 6 and Video S1) and Gamma3 (Figure 7 and Video S2)—was 0.953, with a 95% CI of 0.944–0.960, a Pearson *r* (precision) value of 0.983, and a bias correction factor Cb (accuracy) value of 0.969 (Table 3).

**Table 3:** Concordance between physical and simulated tests, static.

	CCC	95% CI lower	95% CI upper	Pearson *r*	*Cb*
**DHS (*n* = 5)**					
Mean	0.952	0.944	0.960	0.984	0.968
Standard deviation	0.026	0.030	0.022	0.009	0.026
**Gamma3 (*n* = 4)**					
Mean	0.954	0.945	0.961	0.983	0.971
Standard deviation	0.028	0.033	0.024	0.005	0.029
**All static (*n* = 9)**					
Mean	0.953	0.944	0.960	0.983	0.969
Standard deviation	0.025	0.029	0.021	0.007	0.026

**Figure 8:**
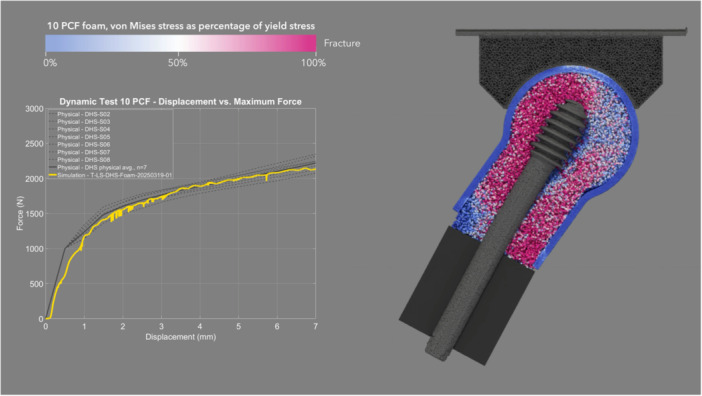
Simulation (yellow) and physical (gray) results of DHS under dynamic loading, cross sectional view through foam proximal femur.

**Figure 9:**
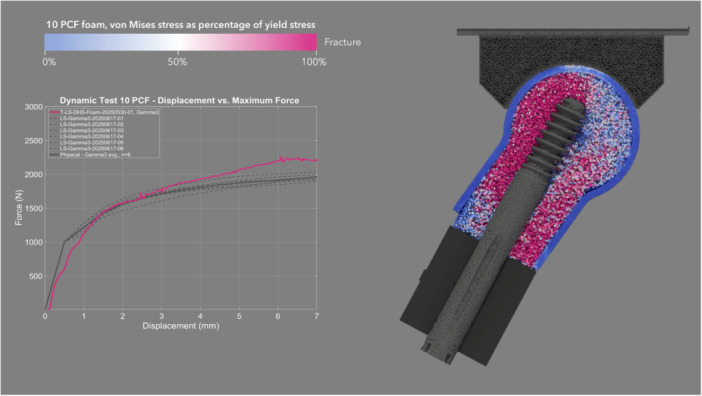
Simulation (pink) and physical (gray) results of Gamma3 under dynamic loading, cross sectional view through foam proximal femur.

**Figure 10:**
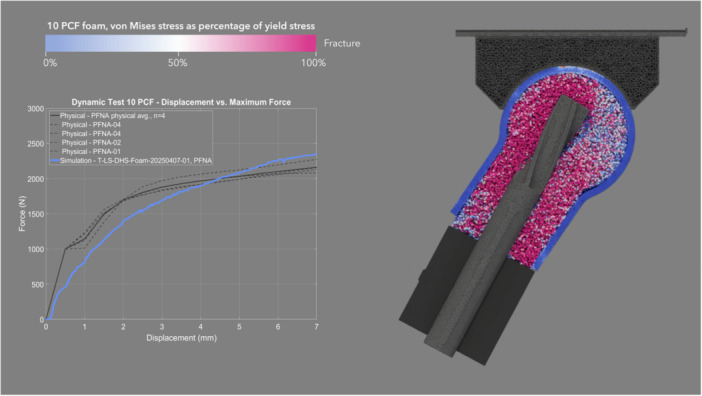
Simulation (blue) and physical (gray) results of PFNA‐II under dynamic loading, cross sectional view through foam proximal femur.

**Figure 11:**
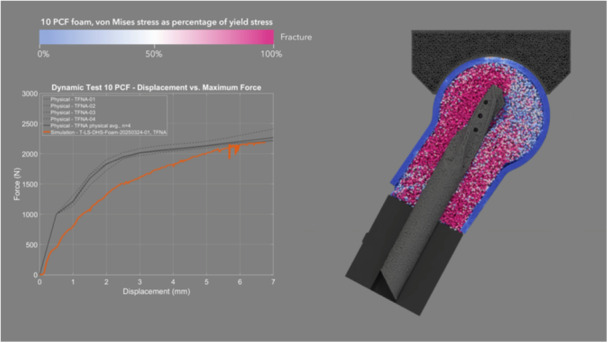
Simulation (orange) and physical (gray) results of TFNA under dynamic loading, cross‐sectional view through foam proximal femur.

The mean CCC between physical and simulated dynamic loading pairs—DHS (Figure 8 and Video S3), Gamma3 (Figure 9 and Video S4), PFNA‐II (Figure 10 and Video S5), and TFNA (Figure 11 and Video S6)—was 0.858, with a 95% CI of 0.837–0.876, a Pearson *r* (precision) value of 0.968, and a bias correction factor Cb (accuracy) value of 0.886 (Table 4).

**Table 4:** Concordance between physical and simulated tests, dynamic.

	CCC	95% CI Lower	95% CI Upper	Pearson *r*	Cb
**DHS (*n* = 7)**					
Mean	0.945	0.934	0.954	0.982	0.962
Standard deviation	0.030	0.035	0.025	0.011	0.024
**Gamma3 (*n* = 6)**					
Mean	0.790	0.762	0.815	0.981	0.805
Standard deviation	0.081	0.089	0.073	0.010	0.082
**PFNA‐II (*n* = 4)**					
Mean	0.863	0.841	0.882	0.947	0.911
Standard deviation	0.019	0.021	0.017	0.014	0.008
**TFNA (*n* = 4)**					
Mean	0.781	0.749	0.809	0.942	0.829
Standard deviation	0.031	0.034	0.028	0.012	0.029
**All dynamic (*n* = 21)**					
Mean	0.858	0.837	0.876	0.968	0.886
Standard deviation	0.086	0.096	0.077	0.021	0.082

**Figure 12:**
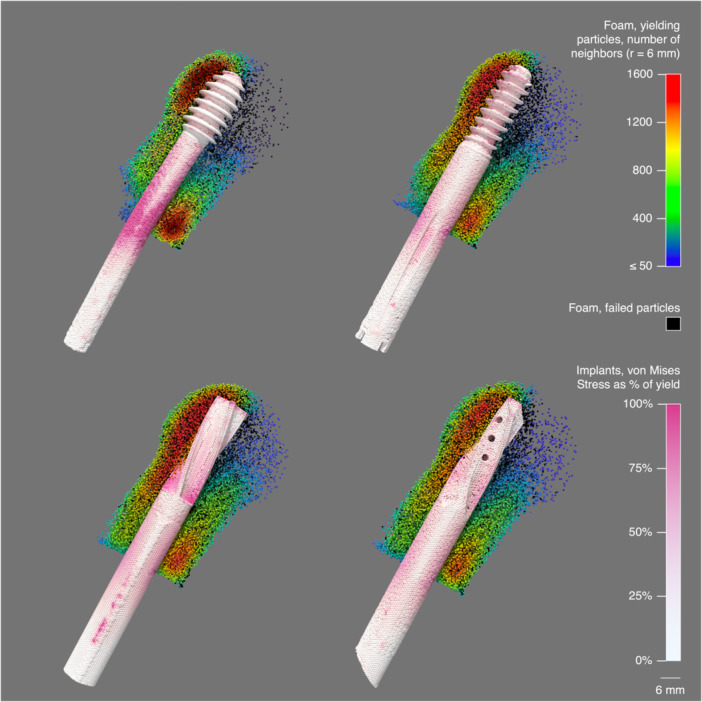
Simulation results of (from top left to bottom right) DHS, Gamma3, PFNA‐II, and TFNA under dynamic loading; cross‐sectional view through proximal femur showing yielding/failed bone foam particles. A density heatmap of yielding foam was created for each device by calculating the number of neighboring yielding particles within a 6 mm radius. Implant von Mises stress is shown as a percentage of the yield (i.e., 800 MPa for DHS in 316LVM steel, 860 MPa for all other devices in titanium alloy).

Under simulated dynamic loading, the volumes of yielding and failed bone foam were 1.695 and 0.224 mL, 1.931 and 0.267 mL, 2.303 and 0.228 mL, and 2.188 and 0.208 mL, for the DHS, Gamma3, PFNA‐II, and TFNA, respectively (Figure 12 and Videos [Link], [Link], [Link], [Link]). Compared to that of the DHS, the volumes of yielding or failed bone foam material were 15%, 32%, and 25% higher for the Gamma3, PFNA‐II, and TFNA, respectively, while the simulated dynamic force at cut‐out was only 2.9%, 10%, and 2.6% higher than that of the DHS, respectively (Table 5).

**Table 5:** Yielding and failed particles during dynamic simulations.

	DHS	Gamma3	PFNA‐II	TFNA
Force at cut‐out (*N*)	2132	2194	2354	2187
Force at cut‐out versus DHS (%)	—	+2.9%	+10%	+2.6%
Yielding particle count	26,490	30,179	35,978	34,188
Failed particle count	3502	4171	3563	3257
Volume of yielding particles (mL)	1.695	1.931	2.303	2.188
Volume of failed particles (mL)	0.224	0.267	0.228	0.208
Volume of yielding or failed particles (mL)	1.919	2.198	2.531	2.396
Volume of yielding or failed particles versus DHS (%)	—	+15%	+32%	+25%

## 4 Discussion

There was no significant difference in the mean force at cut‐out between DHS and Gamma3 under physical static loading. The physical and simulation curves of the static test had high concordance (Table 3). The simulation predicted the physical static force at cut‐out within 0.87% and 3.28% for DHS and Gamma3, respectively (Table 2). The simulated static test showed yielding bone foam particles located primarily superior to the screw, and at the inferior–medial neck region due to varus rotation of the foam femur (Figures 6 and 7). This deformation of the foam was also observed in the physical test.

The mean force at cut‐out was lower in the dynamic test than in the static test for the two devices that were tested in both loading scenarios (DHS and Gamma3). This suggests that applying dynamic cycles with a monotonically increasing load, rather than a static load, attenuated the resistance to implant migration due to the accumulation of damage to the bone foam material over loading cycles. Gamma3 had a significantly lower mean force at cut‐out than the three other devices tested under physical dynamic loading (DHS, PFNA‐II, and TFNA had 13.27%, 10.31%, and 15.72% higher cut‐out force than Gamma3, respectively). The physical and simulation curves of the dynamic test had the highest concordance for DHS (mean CCC = 0.945) and the lowest concordance for TFNA (mean CCC = 0.781) (Table 4). The simulated dynamic test predicted initial migration and the slope of the force–displacement curve more accurately for screw‐type designs rather than blade‐type designs. The simulation predicted the physical dynamic force at cut‐out within 3.92%, 12.00%, 8.93%, and 3.53% for DHS, Gamma3, PFNA‐II, and TFNA, respectively (Table 2).

In the physical dynamic test, both blade‐type devices (PFNA‐II and TFNA) had a higher resistance to initial migration, requiring higher dynamic forces to reach a given displacement, than both screw‐type devices (DHS and Gamma3) (Figure 5). The higher resistance to onset of migration of the blade‐type implants could be due to compaction of the bone foam around the blade during the physical insertion procedure as they are hammered in rather than screwed in. Goffin et al. studied this theory in a finite element computational model and concluded that bone compaction around a PFNA helical blade decreases the risk of cut‐out in very osteoporotic bone (Young's modulus of 533 MPa) but not for denser bone (Young's modulus of 820 MPa) [64]. Other evidence in the literature [65], however, calls into question the relationship between bone compaction during insertion and fixation strength.

As the loading cycles increased in the physical dynamic test, the DHS curves approached the force level of the blade‐type devices at cut‐out, defined as 7 mm of vertical displacement. DHS and Gamma3 had similar resistance to initial migration as shown by the initial slope of their force–displacement curves, but the DHS and Gamma3 physical dynamic curves began to diverge from around 3 mm of displacement with the DHS requiring more force to reach a given displacement than Gamma3. In the dynamic simulations, we can see the stress that develops in the shaft of the DHS is higher than in the other devices (Figure 12 and Supporting Videos). This is likely due to the DHS having the smallest shaft diameter of the devices tested, but slight bending of the DHS shaft may have helped to absorb a portion of the applied load and provided some relief in the bone foam material.

The heatmaps illustrating the density of yielded particles in the simulated dynamic test show a wider distribution of yielded particles along the superior surface of the blades and the long threaded region of Gamma3, whereas the yielded particles are concentrated at the short threaded region of the DHS (Figure 12). This agrees with the pattern of material compaction we observed in x‐rays taken after physical dynamic testing (Figure S3). Simulated testing can provide additional insight into how the applied load is distributed within the surrounding material for different implant designs.

In this study, blade‐type proximal femoral fracture fixation devices had higher resistance to the onset of implant migration in terms of the force required for displacement under dynamic loading in comparison to screw‐type devices. However, as dynamic loading progressed, there was no implant that clearly had superior performance. For elderly patients with poor quality or osteoporotic bone, preventing fixation failure at the bone–implant interface and resistance to initial implant migration is critical, but sustained resistance to further implant migration is also necessary to minimize excessive migration that can lead to life‐threatening fixation failures such as cut‐out and nonunion [66, 67]. Other than predicting the force to initiate migration or to reach cut‐out, other factors may be important for clinical implant selection. Simulation showed that devices with similar force at cut‐out can result in relatively large differences in the volume of damaged bone foam. This suggests that accurate modeling of material damage may provide a useful alternative method for differentiating device performance under dynamic loading. Understanding how an implant stabilizes itself in the bone and how it load shares with the surrounding bone can be critical for implant placement. These additional insights can help a surgeon target fixation in high‐density regions of bone and avoid generating high stress where fracture lines are present.

Our overall findings are similar to other studies reported in the literature, but different studies often report a different device as superior. Sommers et al. used cellular polyurethane foam (36 MPa compressive modulus) to make surrogate specimens of the femoral head and neck, then implants were inserted to a depth of 12.2 mm from the screw tip to femoral head apex and offset 7 mm posteriorly to simulate nonideal placement [52]. The cut‐out resistance in a reduced unstable pertrochanteric fracture model was compared for four different implants: DHS, dynamic helical hip blade, TFN, and Gamma lag screw. The implants were oriented to reflect a 130° femoral neck angle, 16° resultant joint load vector, and 3° offset of the femoral shaft axis from the sagittal plane. The surrogate foam specimens were confined in a stainless steel shell, and dynamic sinusoidal loading was applied. Sommers et al. found that TFN implants sustained statistically significant higher number of cycles to cut‐out compared to the other three implant types and that blade‐type implant designs significantly delayed the onset of migration. In comparison, our present study used a denser and closed‐cell foam, different implant placement in the foam, and a similar but not the same setup and loading profile. Despite this, our physical dynamic results had similar findings to Sommers et al., where TFNA had the highest force to cut‐out, and the blade‐type devices had higher resistance to the onset of implant migration.

Born et al. used cylinders made of 15 PCF solid rigid polyurethane foam (4.9 MPa compressive strength, 123 MPa compressive elastic modulus) and eccentrically positioned implants 5 mm inferior at a tip–apex distance of 45 mm to represent a worst‐case scenario [68]. Four types of hip screws or blades were tested: Gamma3, Gamma3 rotational control (RC), PFN‐A, and TFN. The foam cylinders were surrounded by a steel cup, and a clinical worst‐case scenario of unstable baso‐cervical and trochanteric fracture was reproduced. The gait movement of the hip in the sagittal plane was simulated by an oscillating movement and a hip force was applied axially to reproduce a double peak loading occurring over a cycle of flexion to extension. Migration resistance was highest for Gamma3 RC, followed by Gamma3, PFN‐A, and TFN. Gamma3 migrated predominantly in the cephalad direction, whereas the TFN and PFN‐A helical blades showed a small cephalad migration but migrated markedly in the axial direction into the femoral head. In comparison, our present study used a lower‐density foam, different implant placement in the foam, a similar but not the same setup, and a simpler gait loading profile. Our physical dynamic test resulted in TFNA having the highest force to cut‐out and Gamma3 the lowest, which are opposite results to Born et al. The eccentric screw placement, large TAD, and dynamic multiplanar loading model of Born et al. may explain the different outcome to our study.

A limitation of our study was that the surgical insertion procedures of the implants were not simulated before loading. Instead, the implant models were inserted in the bone foam by removing foam particles intersecting with the implant solid model. Simulations of DHS and Gamma3 are less likely to be affected by insertion, as their implantation procedures involve pre‐tapping of the pilot hole or self‐tapping cutting flutes. For the blade‐type designs, simulating the insertion procedure or modeling postinsertion densification of the foam around the blade may account for the underpredicted force values during the initial loading phase. The hole preparation procedure (e.g., drill dimensions and drill depth) should also be carefully considered in the model setup, as this could affect the fit between the test material and the implant. Another limitation of this study was the relatively small sample size. Future studies should conduct a power analysis prior to testing to determine the sample size needed to detect a clinically meaningful difference between the device groups.

Given that this was a biomechanical study using an idealized bone surrogate material with a steel shell, the results may not translate directly to what is observed in clinical outcomes. Rigid unicellular polyurethane foam is more homogenous and less anisotropic than natural bone, and thus it does not exactly correspond to the mechanics and morphometry of human bone [69, 70]. The current literature is lacking in studies testing whether the fatigue properties of polyurethane foam are representative of natural bone. More sophisticated bone surrogate models (e.g., foam core with epoxy cortical shell) and “population‐specific” synthetic models are being developed and could be a step closer to more realistic modeling of bone geometry and mechanics [71, 72, 73]. Future studies would benefit from testing implants in synthetic bone and computational bone models that represent a range in the target patient population in terms of bone morphometry and distribution of bone mineral density.

## Author Contributions

All authors contributed substantially to data interpretation and manuscript drafting and have reviewed the final submitted manuscript. Erica Ueda Boles, Sloan Kulper, and Christian X. Fang designed the physical experiments. Erica Ueda Boles and Marilyn Janice Oentaryo performed the physical experiments. Erica Ueda Boles and Sloan Kulper designed the numerical models. Sloan Kulper and Katie Whiffin performed the simulations and visualized the simulated data.

## Supporting information

SV‐1‐Static‐DHS‐cropped.

SV‐2‐Static‐Gamma3‐cropped.

SV‐3‐Dynamic‐DHS‐cropped.

SV‐4‐Dynamic‐Gamma3‐cropped.

SV‐5‐Dynamic‐PFNA‐II‐cropped.

SV‐6‐Dynamic‐TFNA‐cropped.

SV‐7‐Dynamic‐yielding‐failed‐DHS‐box.

SV‐8‐Dynamic‐yielding‐failed‐Gamma3.

SV‐9‐Dynamic‐yielding‐failed‐PFNA.

SV‐10‐Dynamic‐yielding‐failed‐TFNA‐box.

JOR ‐ Manuscript ‐ Revision Draft ‐ 20251219 ‐ Table S‐1.

JOR ‐ Manuscript ‐ Revision Draft ‐ 20251219 ‐ Table S‐2.

supmat.
