# Supplementary material for: Comparison of the Dynamic Cut‐Out Failure Modes of Common Proximal Femoral Fixation Devices Using a Mesh‐Free Computational Method
Source: J Orthop Res. 2026 Feb 10;44(2):e70159. doi: 10.1002/jor.70159 (PMC12890569; doi:10.1002/jor.70159)
Supplement: Supplementary file 11 — JOR ‐ Manuscript ‐ Revision Draft ‐ 20251219 ‐ Table S‐1. [file JOR-44-0-s013.docx]

**Table S-1**. Lin’s CCC analysis comparing physical and simulated force displacement curves of 10 PCF foam

| **Variables** | **Average Physical Trials (n = 6) vs. Simulation (8.5x correction coefficient)** |
| --- | --- |
| **Sample size (curve data points)** | 474 |
| **Concordance correlation**  **coefficient** | 0.9564 |
| **95% Confidence Interval** | 0.9503 to 0.9618 |
| **Pearson ρ (precision)** | 0.9858 |
| **Bias correction factor C_b_ (accuracy)** | 0.9701 |
