# Supplementary material for: Comparison of the Dynamic Cut‐Out Failure Modes of Common Proximal Femoral Fixation Devices Using a Mesh‐Free Computational Method
Source: J Orthop Res. 2026 Feb 10;44(2):e70159. doi: 10.1002/jor.70159 (PMC12890569; doi:10.1002/jor.70159)
Supplement: Supplementary file 12 — JOR ‐ Manuscript ‐ Revision Draft ‐ 20251219 ‐ Table S‐2. [file JOR-44-0-s008.docx]

**Table S-2**. Lin’s CCC analysis comparing physical and simulated uniaxial foam compression of other foam grades

| **Variables** | **15 PCF (n=6)** | **20 PCF (n=6)** | **40 PCF(n=6)** |
| --- | --- | --- | --- |
| **Sample size (curve data points)** | 442 | 430 | 348 |
| **Concordance correlation**  **coefficient** | 0.9909 | 0.9883 | 0.8714 |
| **95% Confidence Interval** | 0.9895 to 0.9920 | 0.9860 to 0.9902 | 0.8542 to 0.8868 |
| **Pearson ρ (precision)** | 0.995 | 0.9924 | 0.9801 |
| **Bias correction factor C_b_ (accuracy)** | 0.9958 | 0.9958 | 0.8891 |
