# Supplementary material for: Comparison of the Dynamic Cut‐Out Failure Modes of Common Proximal Femoral Fixation Devices Using a Mesh‐Free Computational Method
Source: J Orthop Res. 2026 Feb 10;44(2):e70159. doi: 10.1002/jor.70159 (PMC12890569; doi:10.1002/jor.70159)
Supplement: Supplementary file 13 — supmat. [file JOR-44-0-s004.docx]

**SUPPLEMENTARY MATERIAL**

**Metallic material model validation via three-point bending**

A series of three-point bending mesh-free simulations in the manner of ASTM F1264^77^ were performed to validate the modeling approach used for metallic components of the current study (**Figure S-1**). A Ti6Al4V rod of known dimensions and force-displacement curve results from the literature^78^ was modeled in CAD. This model was then meshed in Salome v.9.13, and volumetric point clouds were generated in Alfonso v.Y-20240311 using mesh-fitting localized refinement to achieve smooth bearing surfaces at 200 µm, 400 µm, and 800 µm particle resolutions (nominal particle cross-sections of 40 – 160, 80 – 320, and 160 – 640 µm, respectively) to test for convergence. Loading was applied at a uniform rate of 2 m/s. Force-displacement curves for all three modeling resolutions generally agreed well with the literature and suggested acceptable convergence for the level of precision of the present study.

| 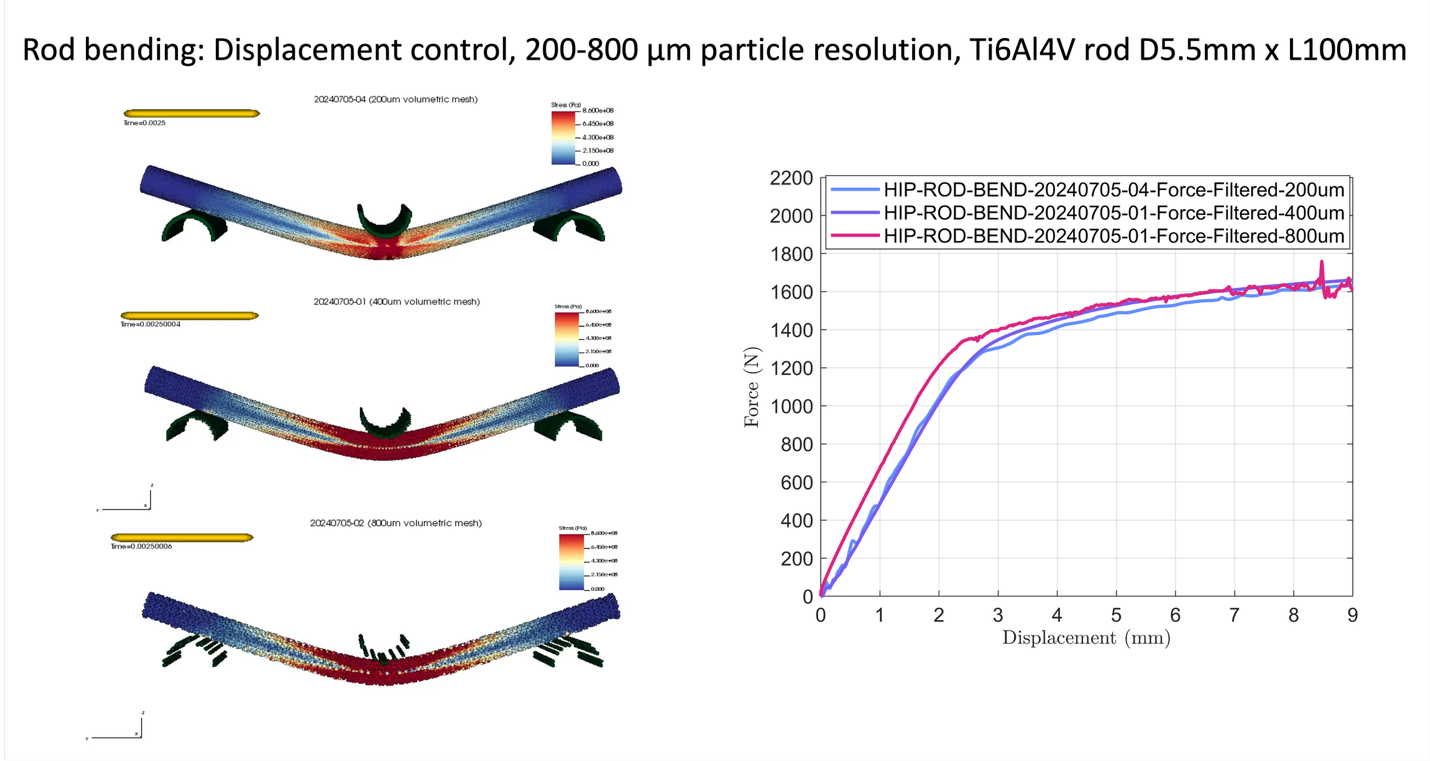  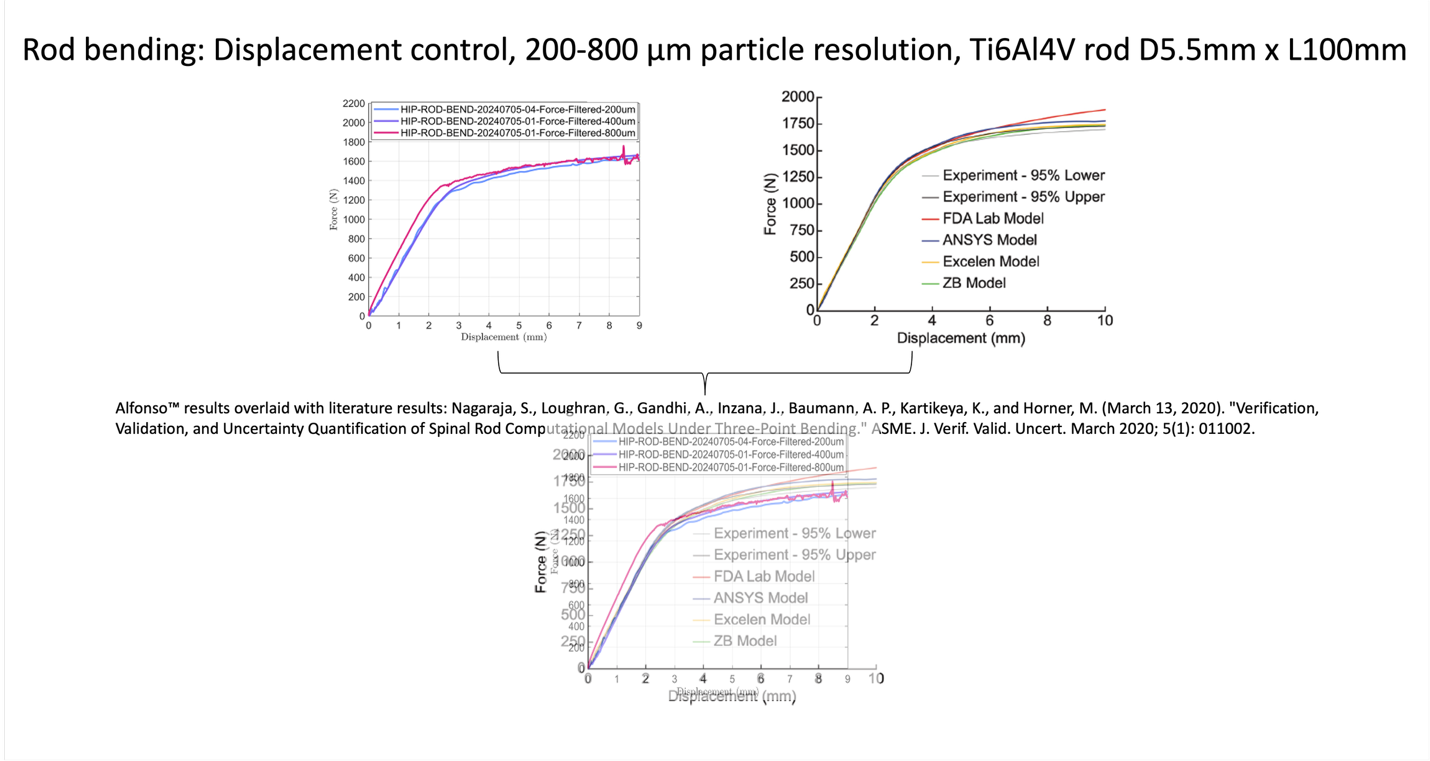  **Figure S-1**. Convergence study, three-point bending simulation of Ti6Al4V rod of known mechanical properties |
| --- |

**Bone foam material model validation via uniaxial compression**

An iterative process was used to determine the appropriate material properties to assign to mesh-free particles of solid polymer such that simulated uniaxial compression results of the overall porous structure agreed with physical testing. Using an MTS 858 Mini Bionix (MTS, Inc., Minnesota, USA), we performed a small series (n=6) of physical uniaxial compression tests of 10 PCF solid rigid polyurethane foam cubes (10 mm on a side) to 10% original height while recording force applied by the loading platen. We then prepared porous mesh-free particle models of the foam cubes using the same process described in the methods section of this study (400 µm resolution) and performed simulated uniaxial compression.*^79^* Using the stated material properties from the manufacturer as a starting point, several material property correction coefficients were attempted (**Figure S-2**). Through iteration, a correction coefficient of 8.5x (applied uniformly to elastic modulus, yield stress, and ultimate stress) was found to achieve good agreement with physical experimental results of uniaxial foam compression.

Both the physical and simulated curves have the typical three distinct regions corresponding to the behaviors of the PU foam’s cell walls during compression (**Figure S-2**). ^80^ Firstly, the linear elastic phase up to 1 mm of the displacement where the cell walls in the PU foam started to bend due to the small increases of loads. Secondly, the long plateau collapse phase that occupied the majority of the load-displacement curve where the cell walls buckled, yielded, or fractured. Lastly, towards the end of the load-displacement curve, the densification phase was displayed as the cell walls eventually compacted and crushed together. Quantitative analysis using Lin’s CCC (**Table S-1**) shows that the concordance between the physical and simulated compression tests of 10 PCF foam using an 8.5x correction coefficient is above 0.95, indicating excellent agreement.^81^ This approach was repeated for other foam grades (15, 20, 40 PCF) (**Table S-2**), also achieving a high degree of concordance with physical tests.

| 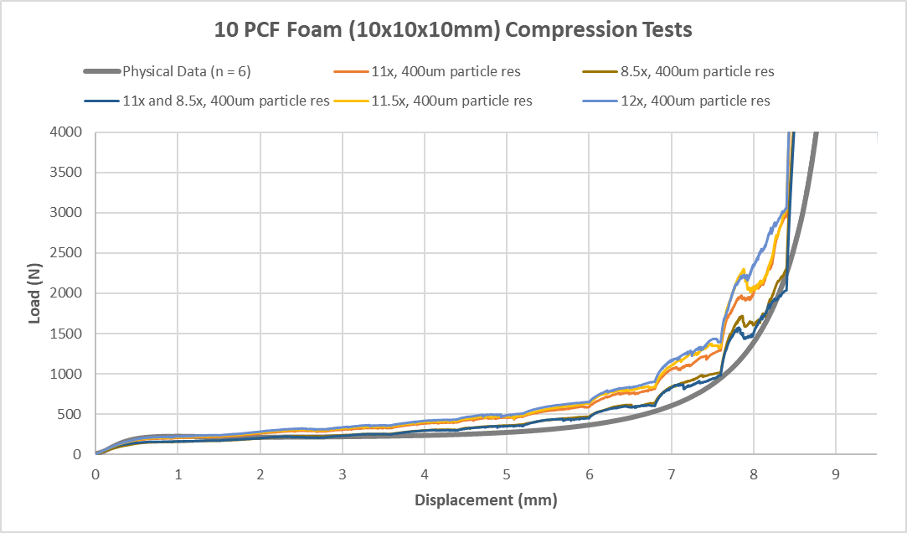 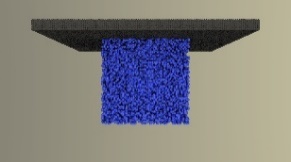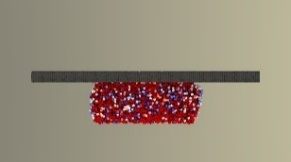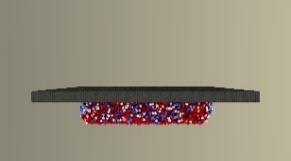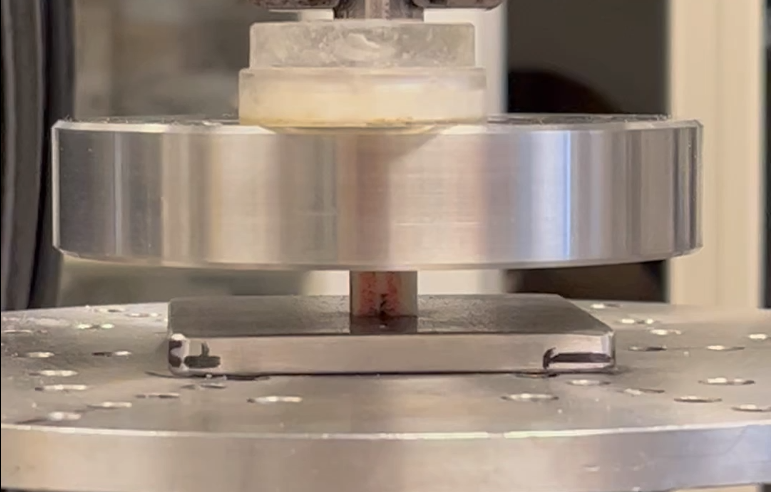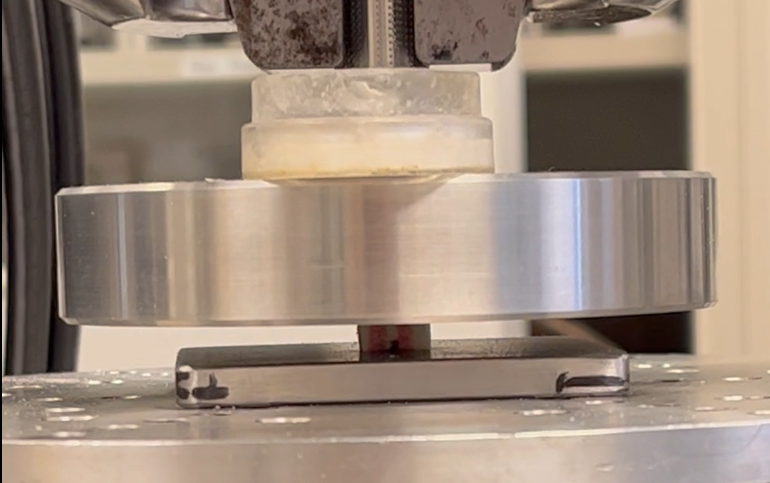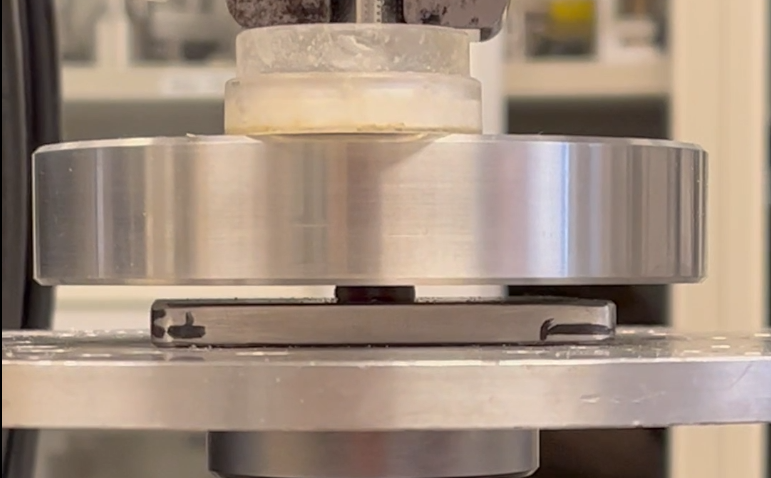 **Figure S-2**. Physical and simulated results of uniaxial foam compression study of 10 PCF solid rigid polyurethane foam cubes (10x10x10 mm) |
| --- |

**Table S-1**. Lin’s CCC analysis comparing physical and simulated force displacement curves of 10 PCF foam

| **Variables** | **Average Physical Trials (n = 6) vs. Simulation (8.5x correction coefficient)** |
| --- | --- |
| **Sample size (curve data points)** | 474 |
| **Concordance correlation**  **coefficient** | 0.9564 |
| **95% Confidence Interval** | 0.9503 to 0.9618 |
| **Pearson ρ (precision)** | 0.9858 |
| **Bias correction factor C_b_ (accuracy)** | 0.9701 |

**Table S-2**. Lin’s CCC analysis comparing physical and simulated uniaxial foam compression of other foam grades

| **Variables** | **15 PCF (n=6)** | **20 PCF (n=6)** | **40 PCF(n=6)** |
| --- | --- | --- | --- |
| **Sample size (curve data points)** | 442 | 430 | 348 |
| **Concordance correlation**  **coefficient** | 0.9909 | 0.9883 | 0.8714 |
| **95% Confidence Interval** | 0.9895 to 0.9920 | 0.9860 to 0.9902 | 0.8542 to 0.8868 |
| **Pearson ρ (precision)** | 0.995 | 0.9924 | 0.9801 |
| **Bias correction factor C_b_ (accuracy)** | 0.9958 | 0.9958 | 0.8891 |

**X-rays of physical dynamic testing samples**

| **Device** | **Pre-test** | **Post-test** |
| --- | --- | --- |
| DHS | 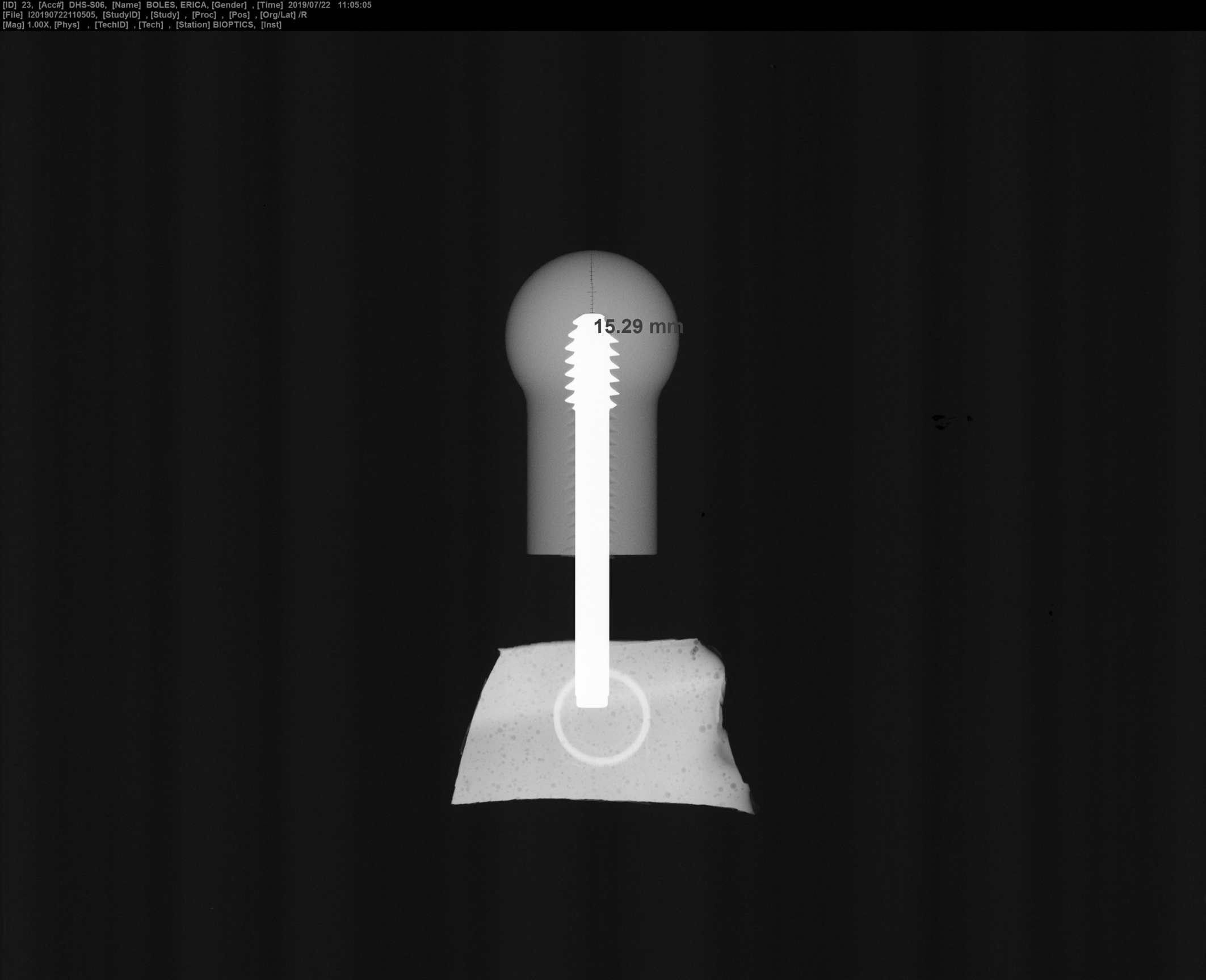 | 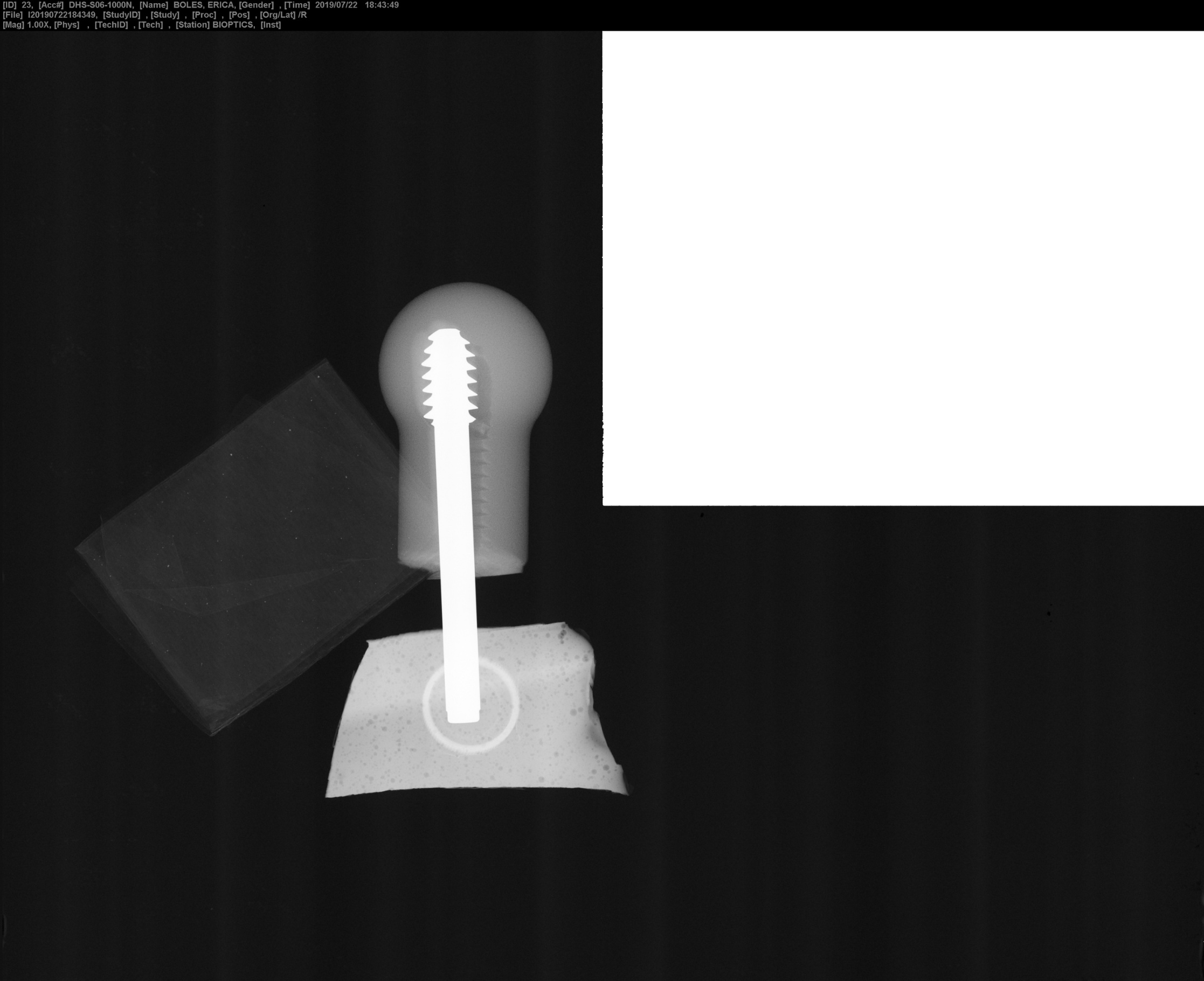 |
| Gamma3 | 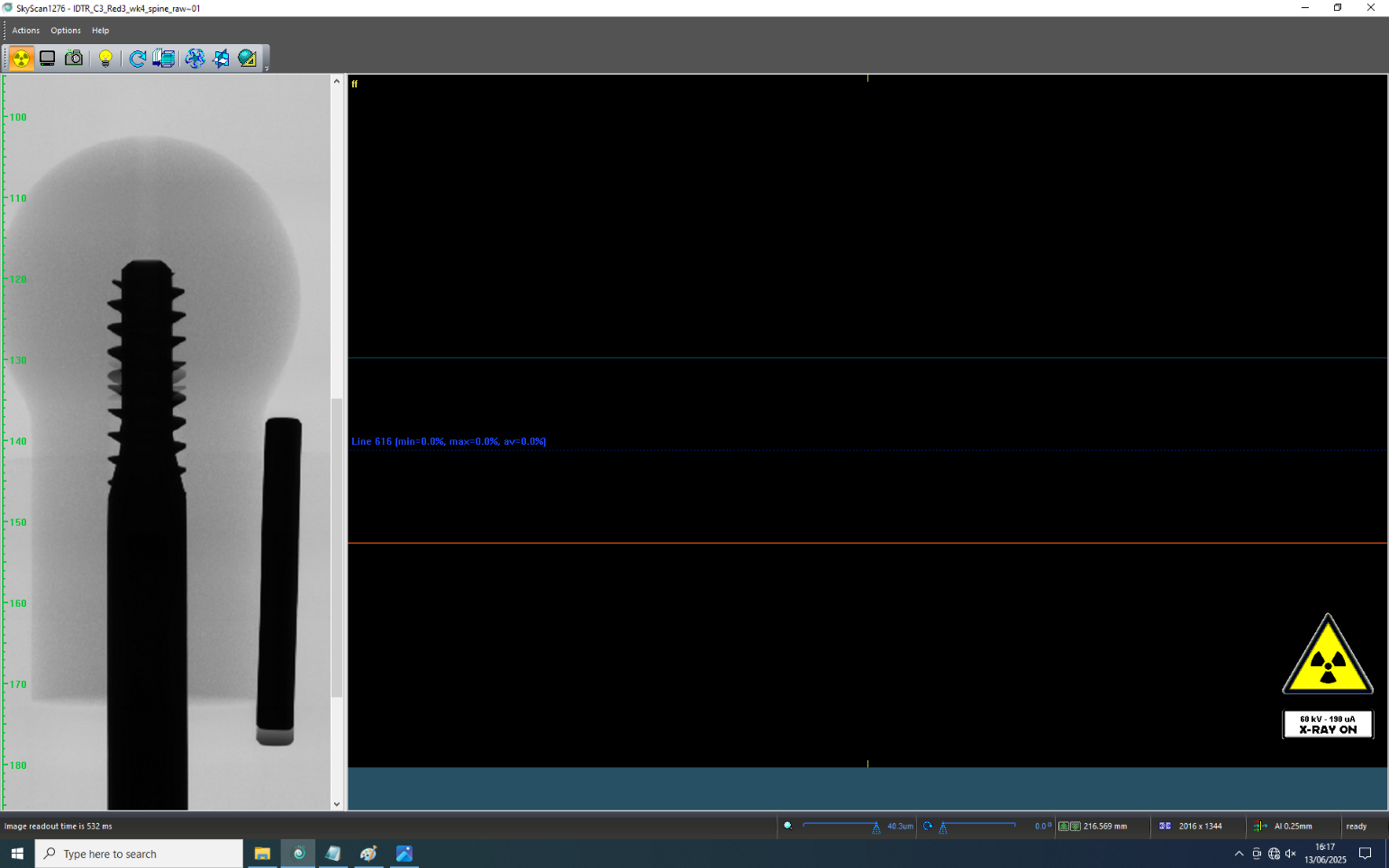 | 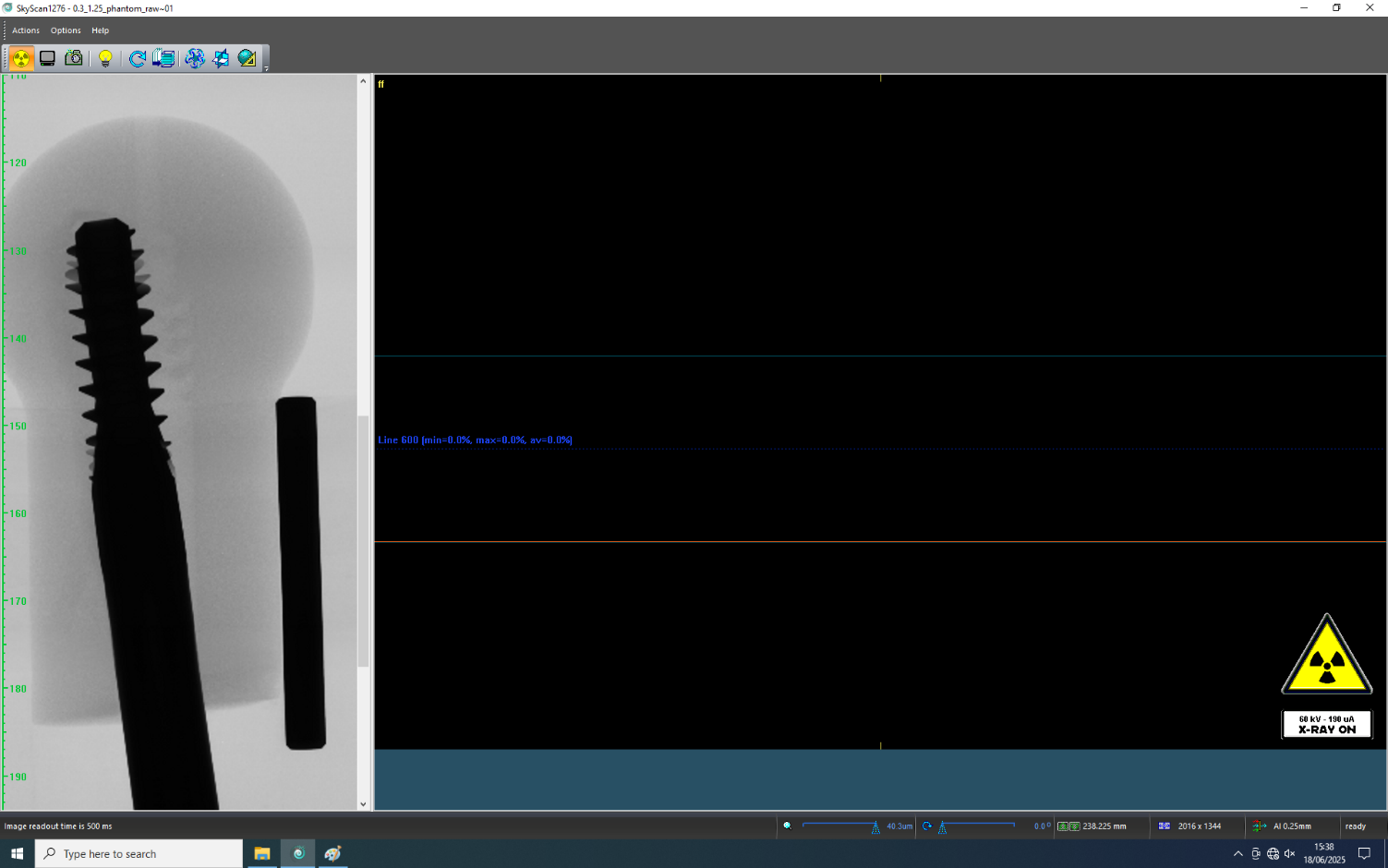 |
| PFNA | 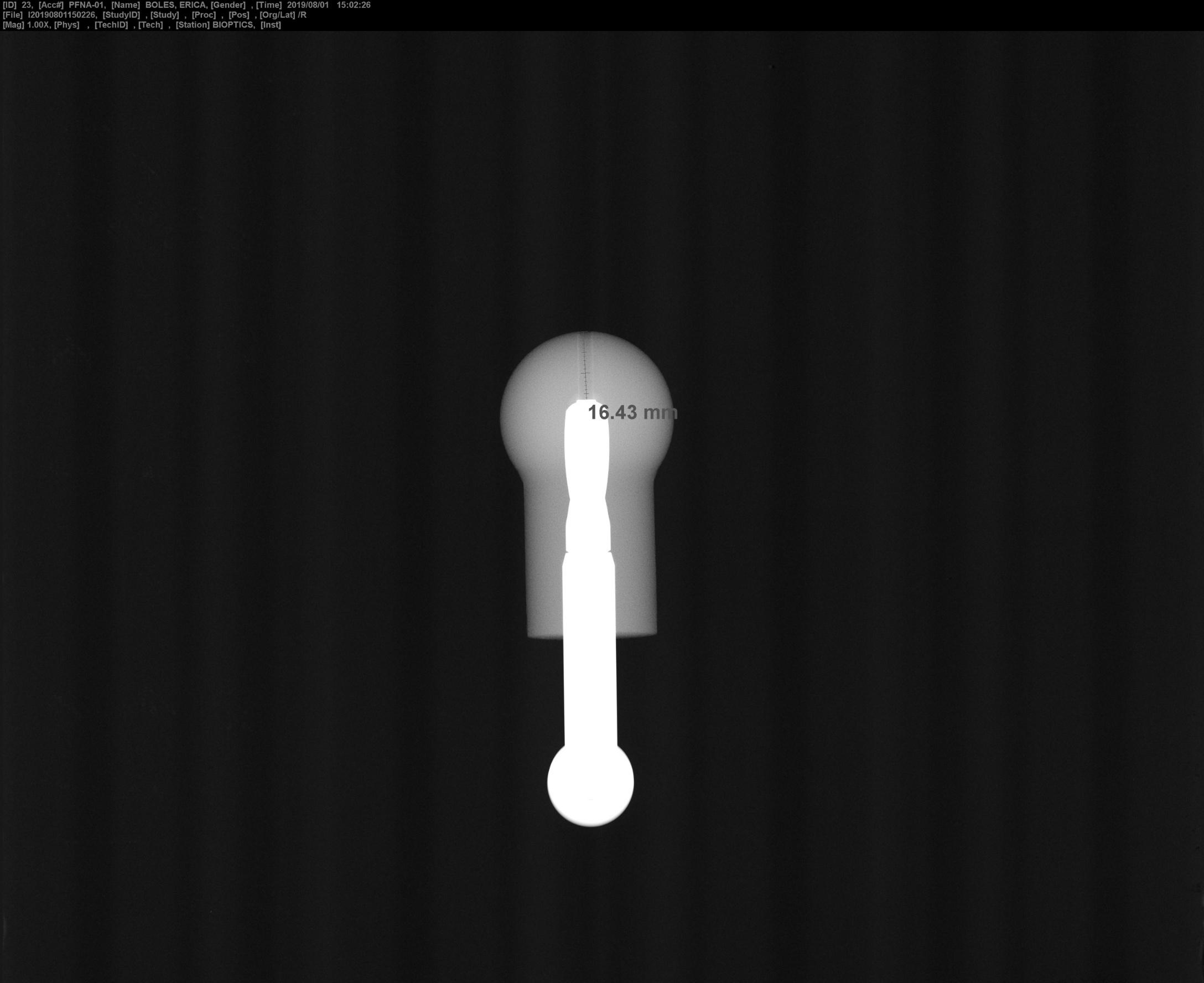 | 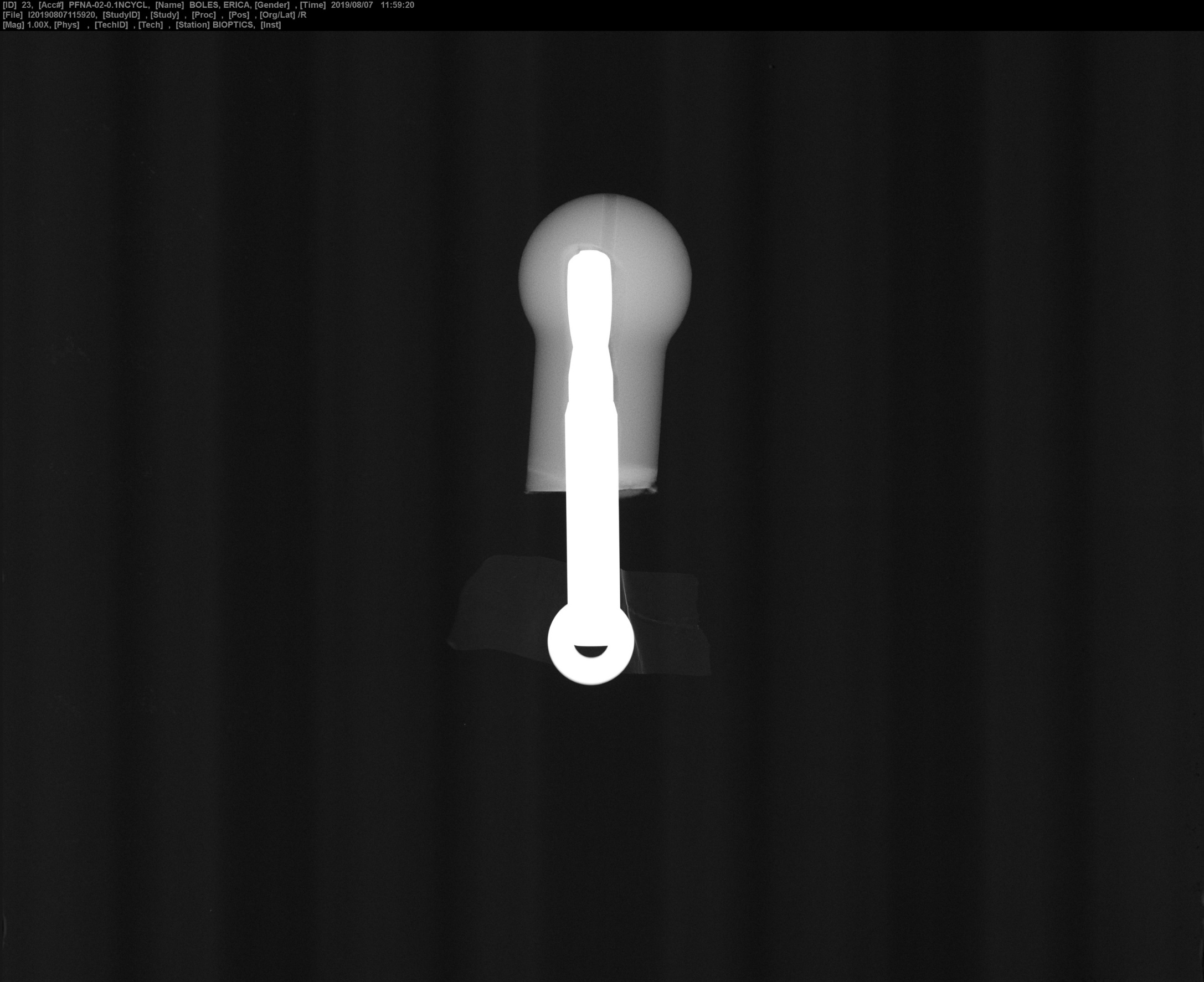 |
| TFNA | 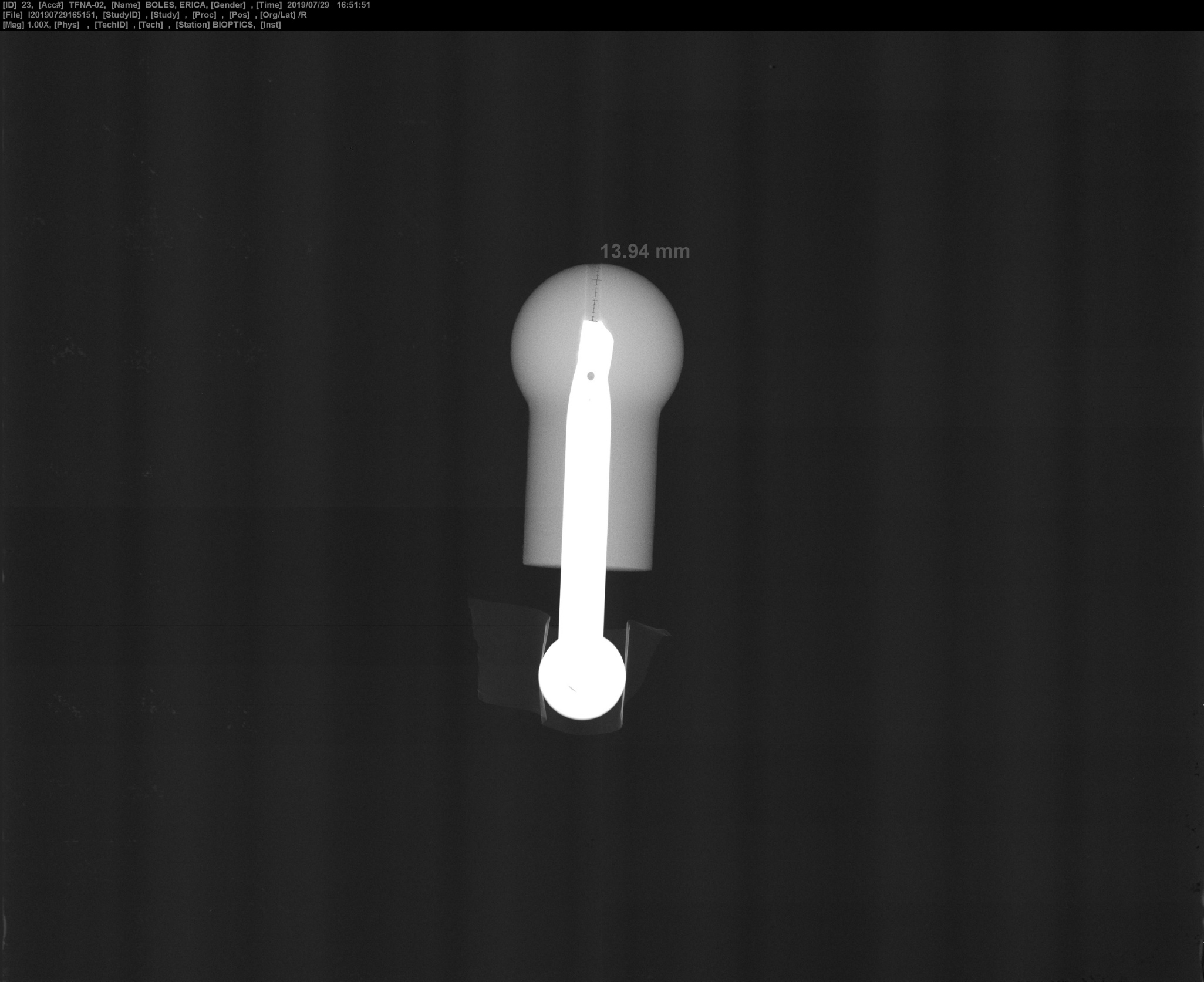 | 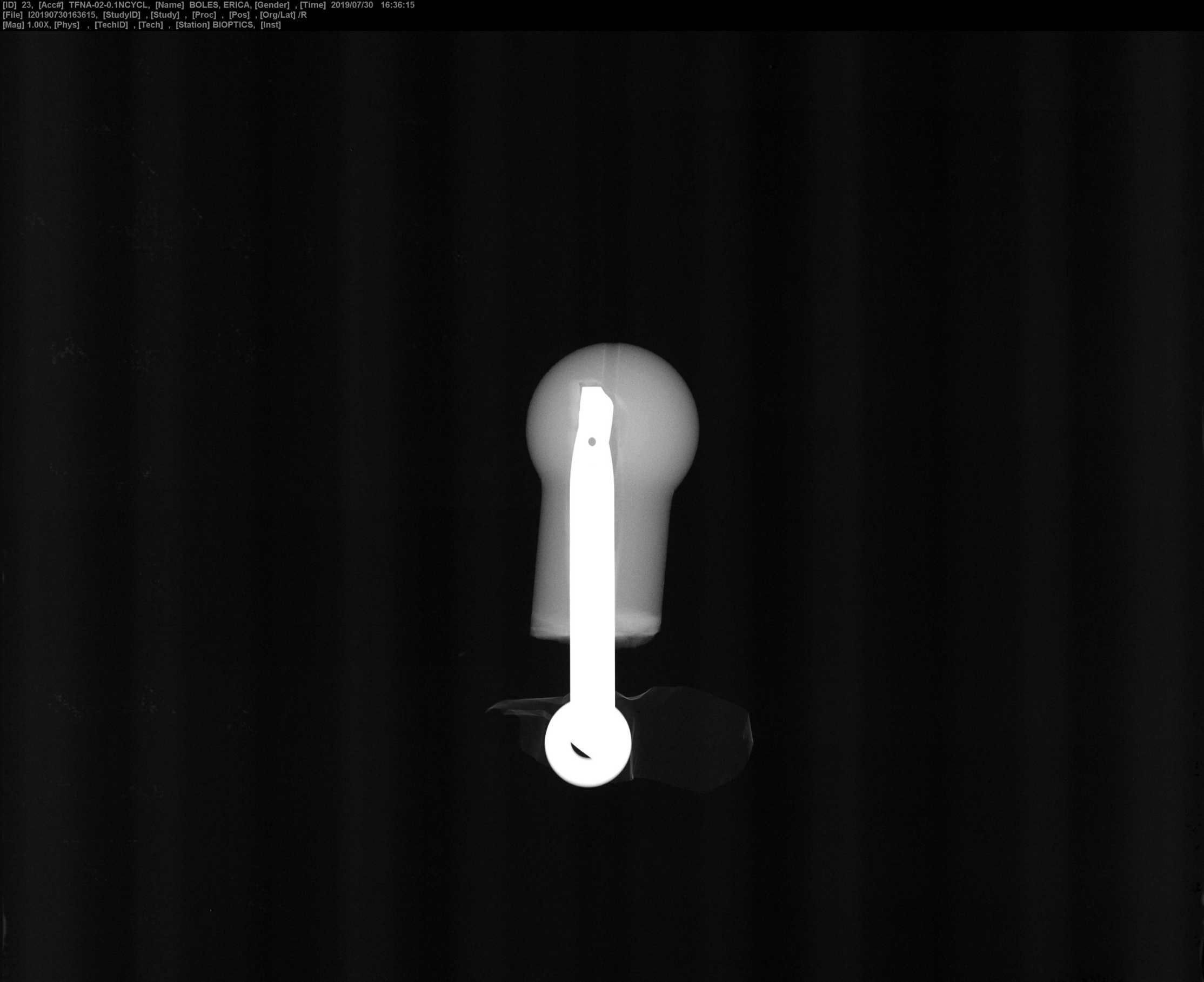 |
| **Figure S-3**. Representative x-ray images in the anterior–posterior view of samples before and after physical dynamic testing. (Only a microCT scan was available for Gamma3.) | | |
